# Drug conjugated nanoparticles activated by cancer cell specific mRNA

**DOI:** 10.18632/oncotarget.9430

**Published:** 2016-05-18

**Authors:** Nathan P. Gossai, Jordan A. Naumann, Nan-Sheng Li, Edward A. Zamora, David J. Gordon, Joseph A. Piccirilli, Peter M. Gordon

**Affiliations:** ^1^ Department of Pediatrics, Division of Pediatric Hematology and Oncology, University of Minnesota, Minneapolis, MN, USA; ^2^ University of Minnesota Masonic Cancer Center, Minneapolis, MN, USA; ^3^ Department of Pediatrics, Division of Pediatric Hematology/Oncology, University of Iowa, Iowa City, IA, USA; ^4^ Department of Biochemistry and Molecular Biology, University of Chicago, Chicago, IL, USA; ^5^ Department of Chemistry, University of Chicago, Chicago, IL, USA

**Keywords:** gold nanoparticles, drug delivery, leukemia, molecularly targeted therapy, anti-sense

## Abstract

We describe a customizable approach to cancer therapy in which a gold nanoparticle (Au-NP) delivers a drug that is selectively activated within the cancer cell by the presence of an mRNA unique to the cancer cell. Fundamental to this approach is the observation that the amount of drug released from the Au-NP is proportional to both the presence and abundance of the cancer cell specific mRNA in a cell. As proof-of-principle, we demonstrate both the efficient delivery and selective release of the multi-kinase inhibitor dasatinib from Au-NPs in leukemia cells with resulting efficacy *in vitro* and *in vivo*. Furthermore, these Au-NPs reduce toxicity against hematopoietic stem cells and T-cells. This approach has the potential to improve the therapeutic efficacy of a drug and minimize toxicity while being highly customizable with respect to both the cancer cell specific mRNAs targeted and drugs activated.

## INTRODUCTION

**Figure 1:**
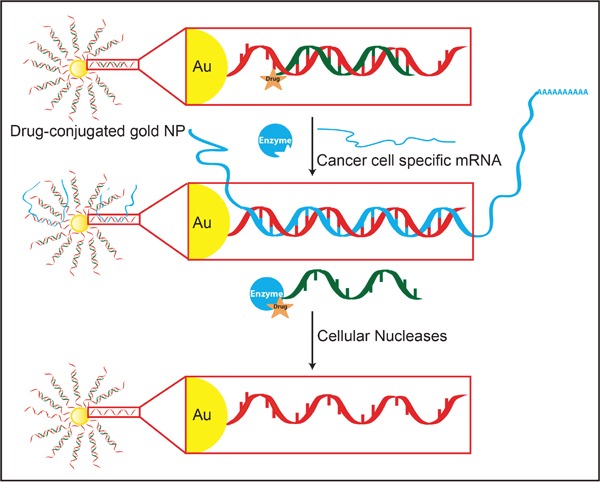
Development of an Au-NP based system for selective drug activation in cancer cells mediated by cancer cell specific mRNA Each gold particle is conjugated to ~150-200 oligonucleotides (red) via a thiol linker. The sequence of the oligonucleotide is complementary (anti-sense) to a mRNA that is either overexpressed in or unique to cancer cells. A shorter, complementary drug-conjugated oligonucleotide (drug-orange; oligonucleotide-green) is annealed to the anti-sense oligonucleotide to generate a drug-DNA Au-NP. After cellular uptake, the targeted mRNA (blue) binds to the complementary DNA sequences linked to the Au-NP. This binding displaces the drug-conjugated oligonucleotide from sequestration to the Au-NP and allows it to inhibit its targeted enzymes. The amount of drug-conjugated oligonucleotide released is proportional to the amount of cancer cell specific mRNA present in the cell. Additionally, mRNAs sequestered by the nanoparticle undergo nuclease degradation.

Despite a rapidly expanding cancer therapy armamentarium many cancers remain incurable and the morbidity and mortality from current therapies remains high. We describe a novel approach to cancer therapy that merges diverse and multi-disciplinary technologies in the areas of drug delivery, pro-drug activation, personalized medicine, and the simultaneous targeting of orthogonal pathways in cancer cells. This approach functionalizes gold nanoparticles (Au-NPs) with short, double-stranded DNA oligonucleotides with sequence corresponding to a gene that is unique to or overexpressed in a cancer cell (Figure 1). Importantly, only the anti-sense strand of the DNA duplex is covalently attached to the gold nanoparticle via a thiol linker at the 3′ terminus of the oligonucleotide. In addition, a molecularly targeted, or cytotoxic, drug is conjugated to the non-covalently linked DNA strand. Drug selection is predicated on known or anticipated efficacy in the targeted cancer and additional chemical and synthetic considerations including that the linkage site occurs at a location within the drug that will not perturb drug binding and efficacy. While nucleic acid functionalized Au-NPs are internalized by many different cell types both *in vitro* and *in vivo* [1], only in cancer cells will the unique, targeted RNA bind to the anti-sense oligonucleotide and displace the drug-conjugated DNA oligonucleotide not covalently linked to the Au-NP. As a binary reaction, the magnitude of drug-conjugated oligonucleotide release correlates with the presence and abundance of the unique RNA. Thus, active (non-sequestered) intracellular drug concentration is enhanced in the cancer cells. Concurrent with drug release, the RNA bound to the Au-NP undergoes degradation by cellular nucleases targeting DNA/RNA hybrids and, therefore, depletes the target cell of a gene required for survival and proliferation [2, 3]. This approach provides a novel targeting opportunity to increase the concentration of free drug in cancer cells relative to normal cells and, therefore, to potentially maximize efficacy and minimize toxicity. This system is potentially tailorable to any cancer for which a unique RNA and appropriate drug exist.

Supporting the feasibility of this approach, sequence-specific, fluorophore-conjugated oligonucleotides attached to Au-NPs have been developed (NanoFlare [3, 4]) and commercialized (SmartFlare; EMD Millipore) as a technology for detecting and measuring RNA levels in living cells. Furthermore, nucleic acid functionalized Au-NPs exhibit additional favorable therapeutic properties including high uptake into diverse cell types that can be in excess of one million nanoparticles per cell, stability in biological environments including resistance to nucleases, minimal cell toxicity, and low immunogenicity [1, 5]. Finally, nucleic acid functionalized Au-NPs delivering siRNA or DNA anti-sense payloads have shown *in vivo* efficacy following intravenous injection against xenotransplanted gastric and brain tumors [6, 7].

## RESULTS

### Conjugation of dasatinib to an oligonucleotide

**Figure 2:**
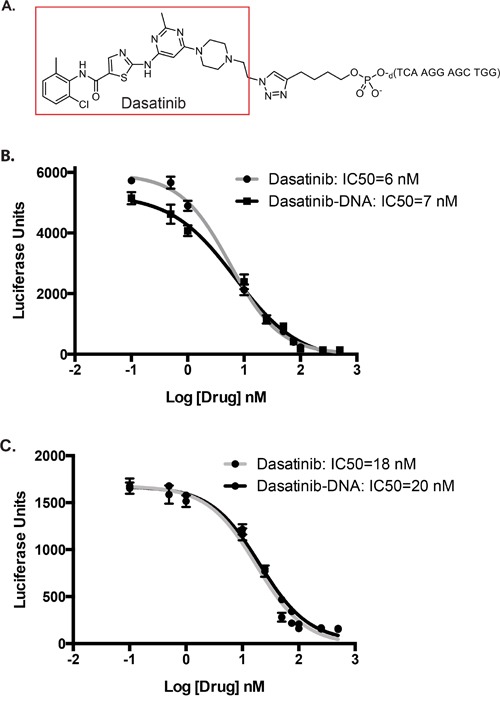
Structure and *in vitro* efficacy of dasatinib conjugated to an oligonucleotide A Structure of dasatinib conjugated to a representative oligonucleotide via copper-catalyzed azide–alkyne cyclo-addition chemistry. **B, C.** SRC (B) and KIT (C) activity were assessed using *in vitro* kinase assays over a range of dasatinib and dasatinib-DNA concentrations. IC50 values were calculated using GraphPad Prism 6.0 and nonlinear regression log(inhibitor) vs. response model. For SRC, R^2^=0.99 (dasatinib) and R^2^=0.98 (dasatinib-DNA). For Kit, R^2^=0.97 (dasatinib) and R^2^=0.98 (dasatinib-DNA).

For proof-of-principle, we selected the drug dasatinib because it is a potent multi-kinase inhibitor [8] (SRC, KIT, BCR/ABL, LYN) and it has been selectively modified at its free hydroxyl position without perturbing its binding affinity to the BCR/ABL kinase [9]. Accordingly, the free hydroxyl on dasatinib was converted to an azide group and then reacted using “click chemistry” [10] with a commercially available oligonucleotide containing a 5′-alkyne functional group to generate a dasatinib-DNA conjugate (Figure 2A and Supplementary Figure S1). As predicted, we found that conjugating an oligonucleotide to dasatinib did not significantly impair its half maximal inhibitory concentration (IC50) for SRC and KIT kinases *in vitro* (Figure 2B, 2C).

### Specificity of Au-NPs for targeted cancer cell specific mRNA

The oligonucleotide sequence conjugated to dasatinib was initially designed to target the human *BIRC5* (*survivin)* mRNA. *BIRC5* mRNA is highly expressed in many cancers relative to differentiated tissues and thus represents an attractive target for this technology [11]. Furthermore, NanoFlare particles designed to target *BIRC5* mRNA have been previously developed and validated [3]. We also designed oligonucleotides to target the breakpoint regions of the t(8;21) (*AML1/ETO*) translocation that occurs with high frequency in acute myeloid leukemia [12]. The breakpoint, or the position in the hybrid mRNA where the sequence shifts from one gene to the other, is unique to only cancer cells and, thus, is also an ideal mRNA target for this approach.

**Figure 3:**
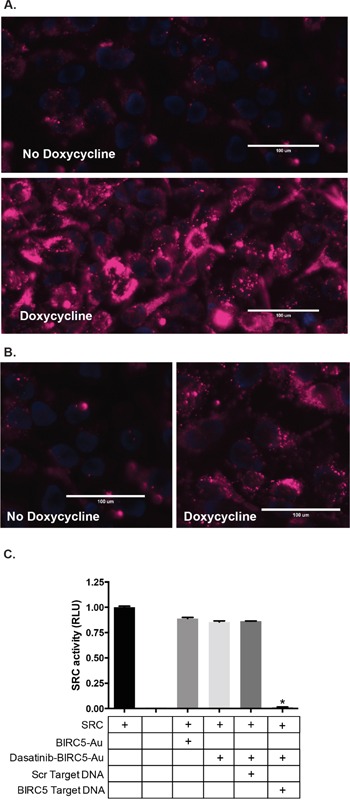
Specificity of Au-NPs for targeted, cancer cell specific mRNAs. A, B Cy5-DNA Au-NPs targeting the human *BIRC5* (A) or *AML1/ETO* (B) mRNAs were added to cells expressing doxycycline-inducible *BIRC5* or *AML1/ETO* mRNA in the presence or absence of doxycycline. For these Au-NPs, the non-covalently linked oligonucleotide contained a Cy5 fluorophore rather than dasatinib. After 24 hours, the cells were stained with Hoechst 33342 dye and assessed by microscopy. Hoechst (blue) and Cy5 (red). Representative images are shown. **C.** Dasatinib-DNA Au-NPs targeting the human *BIRC5* mRNA were added to recombinant SRC kinase in the presence of an excess of either scrambled oligonucleotide or an oligonucleotide mimicking the sequence of human *BIRC5* mRNA. SRC kinase activity was measured using a luciferase-based peptide phosphorylation assay. Data are shown relative to the untreated control and are the mean±s.e.m. from three independent experiments. *, *P* < 0.0001 comparing the untreated control and dasatinib-DNA Au-NPs plus survivin oligonucleotide.

For initial characterization purposes, the non-covalently linked DNA strand contained a terminal Cy5 fluorophore rather than dasatinib (Cy5-DNA). When annealed to its complementary oligonucleotide conjugated to the Au-NP, the fluorophore is quenched by the gold but fluoresces when displaced from the Au-NP by the binding of the target mRNA. Addition of a complementary, but not scrambled, oligonucleotide that mimics the targeted mRNA sequence to the Cy5-DNA Au-NPs increased fluorescence signal *in vitro* and confirmed the specificity of each nanoparticle for its targeted gene (Supplementary Figure S2). We also engineered murine NIH3T3 cells and human HEK293T cells to express human *BIRC5* or *AML1/ETO* mRNA in the presence of doxycycline. Further supporting the specificity of these nanoparticles, after overnight incubation with Cy5-DNA Au-NPs there was increased release of the fluorophore-conjugated DNA oligonucleotide in the presence relative to the absence of doxycycline (Figure 3A, 3B). Together, these experiments demonstrate the selective release of the oligonucleotide from sequestration to the Au-NP in the presence of the cancer cell specific targeted mRNA.

We next synthesized Au-NPs functionalized with *BIRC5* mRNA targeting oligonucleotides in which the non-covalently attached oligonucleotide was conjugated to dasatinib (dasatinib-DNA Au-NP). We tested the specificity of these dasatinib-DNA Au-NPs using the *in vitro* SRC kinase assay (Figure 3C). In the presence of dasatinib-DNA Au-NPs, SRC kinase retains full activity suggesting that the dasatinib-conjugated oligonucleotide is unable to inhibit SRC kinase activity when sequestered to the Au-NP. However, upon the addition of an oligonucleotide mimicking the *BIRC5* mRNA sequence, but not a scrambled control oligonucleotide, SRC kinase activity is significantly attenuated consistent with the sequence specific release of the dasatinib-conjugated oligonucleotide from the Au-NP.

### Highly efficient uptake of Au-NPs by leukemia cells

**Figure 4:**
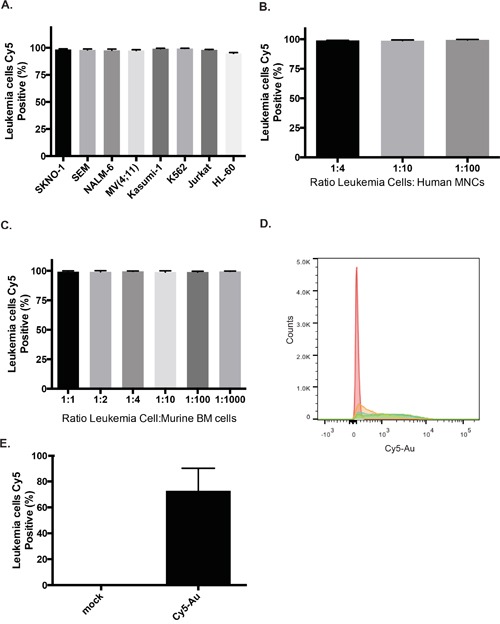
Highly efficient uptake of Au-NPs by leukemia cells **A.** Percentage of leukemia cells containing DNA Au-NPs covalently labeled with Cy5 (see methods) was assessed by flow cytometry after an overnight incubation. Percentages are the mean±s.e.m. from three independent experiments. K562 cells were mixed with either human bone marrow mononuclear cells **B.** or murine bone marrow cells **C.** at the specified ratios and then incubated overnight with DNA Au-NPs covalently labeled with Cy5. Percentages of leukemia cells containing NPs are the mean±s.e.m. from three independent experiments. Flow cytometry histogram **D.** and quantitation **E.** of K562 leukemia cells isolated from subcutaneous flanks tumors that were either injected with Cy5 labeled Au-NPs (blue, green, orange) or un-injected (red).

We utilized human leukemia cells to test the dasatinib-DNA Au-NPs in a more complex cellular environment as high *BIRC5* mRNA expression and dasatinib-sensitive mutations or kinase dependence frequently occur in both chronic and acute leukemia. Efficient uptake (>99%) of fluorescently labeled, nucleic acid conjugated Au-NPs occurred after overnight incubation for all leukemia cell lines tested (Figure 4A). Furthermore, near complete uptake continued to occur in leukemia cells despite being mixed with a 100-1000-fold excess of normal human or mouse bone marrow cells (Figure 4B, 4C), suggesting highly efficient uptake of Au-NPs by leukemia cells. Finally, a single injection of fluorescently labeled Au-NPs into subcutaneous leukemia xenografts also resulted in significant uptake of Au-NPs into leukemia cells (Figure 4D, 4E).

### Dasatinib conjugated Au-NPs inhibit kinase targets in leukemia cells

**Figure 5:**
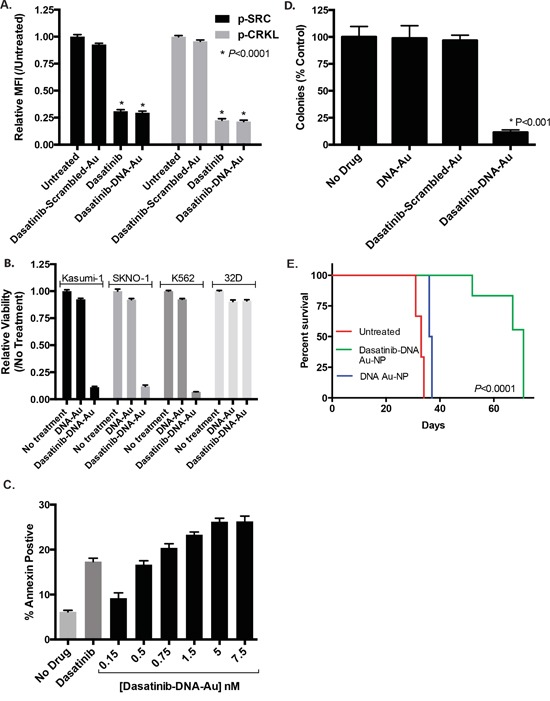
Dasatinib-DNA Au-NPs target human leukemia cells **A.** K562 leukemia cells were treated with either dasatinib or dasatinib-DNA Au-NPs (1 nM) targeting either the human *BIRC5* mRNA or a scrambled control sequence. After 24 hours the cells were fixed/permeabilized, stained for intracellular phospho-SRC or phospho-CRKL, and assessed by flow cytometry. Median fluorescence intensity (MFI) for each condition relative to the untreated control was calculated. *, *P* < 0.0001 for both phospho-SRC and phospho-CRKL when comparing untreated and either dasatinib or dasatinib-DNA Au-NP treated cells. **B.** Dasatinib-sensitive human leukemia cells (SKNO-1, Kasumi-1, and K562) and a dasatinib-insensitive murine hematopoietic cell line (32D) were treated with dasatinib-DNA Au-NPs and after 48 hours proliferation measured using the Cell-TiterGlo luciferase assay. The data are normalized to the untreated controls and are the mean±s.e.m. from three independent experiments. **C.** Induction of apoptosis (Annexin-V positive cells) in K562 leukemia cells 48 hours after treatment with either dasatinib (1 μM) or dasatinib-DNA Au-NPs at the specified concentrations. **D.** Colony formation of K562 leukemia cells treated for 4 hours with dasatinib-DNA Au-NPs, targeting either human *BIRC5* mRNA or a scrambled control, or control NPs lacking dasatinib prior to plating in methylcellulose. Colonies were counted 7 days after seeding 10^3^ cells/plate. Values are expressed as the percentage of colonies from drug treated cells compared to untreated control cells. *, *P* < 0.001 when comparing untreated control cells and cells treated with dasatinib-DNA Au-NPs. **E.** Survival curves for mice intravenously injected with K562 leukemia cells that were either untreated or treated for 4 hours prior to injection with dasatinib-DNA Au-NPs or control DNA-Au-NPs lacking dasatinib. N=3 for untreated and dasatinib-DNA Au-NP treated and N=2 for control NPs lacking dasatinib. *, *P* < 0.0001 when comparing the mice receiving dasatinib-DNA Au-NP treated cells versus untreated leukemia cells.

Following confirmation of efficient uptake of Au-NPs into leukemia cells, we tested dasatinib-DNA Au-NPs with K562 leukemia cells that express *BIRC5* mRNA, harbor a dasatinib-sensitive BCR/ABL translocation, and exhibit high SRC kinase activity. After overnight incubation with dasatinib-DNA Au-NPs, K562 cells were assessed for phospho-SRC and phospho-CRKL levels by flow cytometry. Phosphorylated CRKL, a BCR/ABL adaptor protein, serves as a surrogate of BCR/ABL activity [13]. As shown in Figure 5A, both phospho-CRKL and phospho-SRC levels were significantly diminished in the presence of dasatinib-DNA Au-NPs targeting *BIRC5* mRNA, but not a scrambled control sequence, confirming that dasatinib effectively inhibits its expected targets in a cellular environment. We also tested dasatinib-DNA Au-NPs with the Kasumi-1 leukemia cell line that harbors an activating KIT mutation and expresses *BIRC5* mRNA [14, 15]. It has been previously shown in Kasumi-1 cells that dasatinib prevents the phosphorylation of AKT via inhibition of mutant KIT and SRC kinases [16]. In agreement, dasatinib-DNA Au-NPs decreased phospho-AKT levels with comparable efficacy to dasatinib (Supplementary Figure S3). Furthermore, supporting the bi-functional targeting of these Au-NPs, we also found significantly decreased *BIRC5* mRNA levels after *BIRC5* targeting Au-NP treatment. Although efficient knockdown required a higher concentration of Au-NPs (5 nM) and a longer incubation period than that required for efficient SRC kinase or phospho-CRKL inhibition (Supplementary Figure S4). As previously shown, *BIRC5* mRNA knockdown was enhanced by partial substitution of the DNA backbone with locked nucleic acids and likely occurs via a mechanism analogous to anti-sense technologies [17].

### Dasatinib conjugated Au-NPs diminish the viability of leukemia cells

We next examined the effect of the dasatinib-DNA Au-NPs on leukemia cell proliferation and viability. As shown in Figure 5B, dasatinib-DNA Au-NPs targeting the human *BIRC5* mRNA, but not a scrambled control, significantly inhibit the proliferation of dasatinib-sensitive leukemia cell lines. In contrast, there was minimal effect on 32D cells, a murine hematopoietic cell line lacking human *BIRC5* mRNA. Consistent with this result, sub-nanomolar concentrations of dasatinib-DNA Au-NPs increased apoptosis in K562 leukemia cells as evidenced by annexin-V staining (Figure 5C). Citrate-capped Au-NPs, lacking any nucleic acid functionalization, had no effect on leukemia cell proliferation or apoptosis (Supplementary Figure S5A & B). As illustrated in Figure 5D, dasatinib-DNA Au-NP treatment also strongly reduced the ability of K562 cells to form colonies in methylcellulose, indicating that the dasatinib-DNA Au-NPs inhibit the proliferative potential of leukemia colony-forming cells. Control Au-NPs, either lacking dasatinib or with a scrambled targeting sequence, had no significant effect on K562 colony formation. Finally, treatment of K562 cells with dasatinib-DNA Au-NPs for 4 hours prior to intravenous injection into NSG mice significantly delayed leukemia development (Figure 5E).

### Dasatinib conjugated Au-NPs reduce toxicity against CD34 cells and T-cells

**Figure 6:**
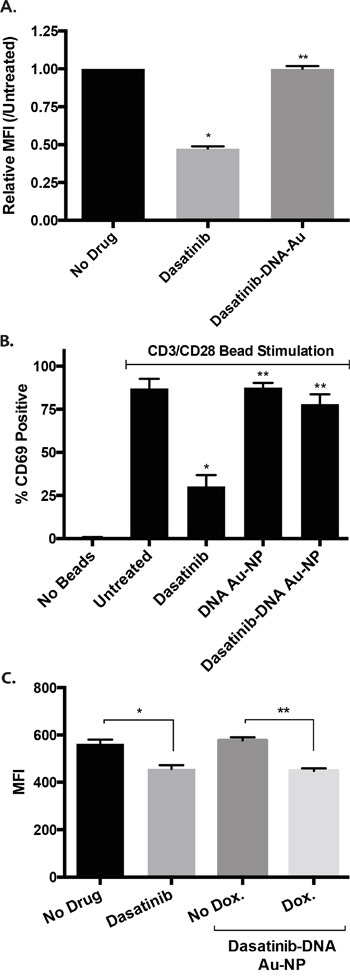
Dasatinib-DNA Au-NPs exhibit less toxicity than dasatinib alone against human CD34 and T-cells **A.** Stimulated (SCF, FLT-3 ligand, thrombopoietin) human CD34 cells were treated with either dasatinib or dasatinib-DNA Au-NPs. After 24 hours, the cells were fixed/permeabilized, stained for intracellular phospho-SRC, and median fluorescent intensity (MFI) assessed by flow cytometry. The MFI values are normalized to the untreated controls and are the mean±s.e.m. from three independent experiments. **, *P* not significant. *, *P* < 0.001 comparing untreated and dasatinib treated cells. **B.** Human CD3+ T-cells were stimulated with anti-CD3/anti-CD28 beads in the absence of any drug or in the presence of either dasatinib or dasatinib-DNA Au-NPs. After 24 hours, T-cell activation was assessed by CD69 staining and flow cytometry. Data are the mean±s.e.m. from three independent experiments. *, *P* < 0.01 and **, *P* not significant when compared to untreated cells. **C.** Dasatinib-DNA Au-NPs targeting the human *BIRC5* gene were added to murine NIH3T3 cells expressing doxycycline-inducible human *BIRC5* mRNA in the presence or absence of doxycycline. After 24 hours, the cells were fixed/permeabilized, stained for intracellular phospho-SRC, and median fluorescent intensity (MFI) assessed by flow cytometry. The MFI values are the mean±s.e.m. from three independent experiments. *, *P* < 0.001 and **, *P* < 0.0001.

Dasatinib inhibits the normal functions of a variety of hematopoietic lineages including CD34 stem cells [18], T lymphocytes [19, 20], NK cells [21], platelets [22], neutrophils [23], and osteoclasts [24]. Based on the selective release of the dasatinib in cancer cells, we predicted that dasatinib-DNA Au-NPs should also exhibit less toxicity than dasatinib against cells with less of the targeted mRNA. In support of this, dasatinib, but not dasatinib-DNA Au-NPs, significantly diminished phospho-SRC levels in human CD34 cells (Figure 6A). Similarly, dasatinib, but not dasatinib-DNA Au-NPs, inhibited T-cell activation by CD3/CD28-beads (Figure 6B). Of note, we observed less avid uptake of Au-NPs in CD34 cells and a subset of T-cells (CD8+) relative to other cell lines tested (Supplementary Figure S6). This diminished Au-NP uptake as well as the sequestration of the dasatinib-conjugated oligonucleotide to the Au-NP in the absence of target mRNA likely both contribute to the diminished toxic effect of dasatinib-DNA Au-NPs on T-cells and CD34 cells. To control for the different Au-NP uptake exhibited by different cells types, we again utilized murine NIH3T3 cells engineered to express human *BIRC5* mRNA in the presence of doxycycline. In this system, dasatinib-DNA Au-NPs decreased phospho-SRC levels in the presence, but not the absence, of doxycycline induced *BIRC5* mRNA expression (Figure 6C). These data also suggest that this approach may be extendable to more toxic drugs than dasatinib.

## DISCUSSION

We have developed a novel therapeutic approach to dramatically increase the concentration of free drug in cancer cells relative to normal cells and, therefore, maximize efficacy and minimize toxicity. While we demonstrated feasibility and efficacy using dasatinib, one of the strengths of this approach is that we envision it to be highly customizable with respect to both the genes targeted and drugs activated. This technology can be tailored to a particular type of cancer by identifying a unique cancer cell specific RNA and appropriate drug. Examples of molecularly targeted drugs that have been successfully conjugated to other biomolecules without diminishing drug efficacy and could, in principle, be utilized in this approach include nilotinib, midostaurin, sunitinib, SN-38, tozasertib, gefitinib, bosutinib, orantinib, olaparib, and purvalanol B [25]. Furthermore, gold nanoparticles can be concomitantly functionalized with multiple drug-conjugated oligonucleotides or biological groups (proteins, siRNA, molecular beacons) and thus could deliver multiple payloads targeting orthogonal cancer pathways. Further functionalizing the drug-conjugated nanoparticles with cancer cell specific antibodies [26] or aptamers [27] may allow for more targeted cancer cell uptake. Additional selective targeting and optimized Au-NP delivery may also be obtained by capitalizing on the enhanced permeability and retention (EPR) effect in solid tumors. The EPR effect is the property by which nanoparticles accumulate in tumors more than in normal tissues secondary to perturbations in tumor vasculature [28]. In support of this, intravenously administered nucleic acid functionalized gold nanoparticles (siRNA and molecular beacons) accumulated in xenografted murine solid tumors and caused tumor regression with otherwise minimal toxicity [6, 7]. In summary, we anticipate that with further optimization this approach may represent a novel and customizable therapy for cancer with the potential to improve the therapeutic efficacy of a drug and minimize toxicity.

## MATERIALS AND METHODS

### Oligonucleotides & reagents

Unmodified, 3′-thiol modified, 5′-hexynyl (alkyne) modified, and fluorophore-conjugated oligonucleotides (Supplementary Table 1) were obtained from IDT and purified as recommended. Oligonucleotides containing locked nucleic acids were obtained from Exiqon. 3′-thiol modified oligonucleotides were deprotected with 100 mM DTT for 1.5 hours at room temperature and then purified using a NAP-5 column (GE Healthcare). Dasatinib was from LC Laboratories. Citrate stabilized, 15 nm Au colloids were obtained from Ted Pella. Cytokines were from PeproTech.

### Oligonucleotide functionalized gold nanoparticle synthesis

Oligonucleotide modified gold nanoparticles were prepared according to literature procedures [4, 29]. In brief, deprotected alkylthiol-terminated oligonucleotides (IDT; 5 μM) were combined with citrate-capped 15-nm gold particles (Ted Pella; 15 nM diameter), 10 nM Na-Phosphate pH 8.0, and 0.01% SDS and incubated for 20-60 minutes at room temperature with gentle rocking. Sodium chloride was then added every 20 minutes in 50 mM increments to achieve a final NaCl concentration of 500 mM. The SDS and Na-Phosphate buffer concentrations were maintained at 0.01% and 10 mM, respectively. The particles were then sonicated for 10 seconds and shaken at room temperature overnight. The DNA conjugated Au-NPs were purified from unattached oligonucleotides by centrifugation at 14,000 rpm for 25 minutes x 3 and re-suspended in phosphate buffered saline (PBS). Next, the complementary oligonucleotide (either dasatinib or fluorophore conjugated), at ~100-fold excess, was hybridized to the purified DNA-conjugated Au-NPs by heating the mixture to 80°C and then slowly cooling to 4°C over several hours in a PCR machine. The resulting Au-NPs were purified by centrifugation at 14,000 rpm for 25 minutes x 3 at 4°C, suspended in PBS, and stored at 4°C. Au-NP concentration was determined by measuring absorbance at 520 nm [29].

Au-NPs in which the fluorophore is covalently attached to the Au-NP (i.e. for the experiments shown in Figure 4A) were either purchased (Cy5 or Cy3 Uptake Control SmartFlare, EMD Millipore) or were synthesized by two different approaches. First, Au-NPs were functionalized as described above with an oligonucleotide containing both a 3′-thiol linker and a 5′-Cy5 modification. Alternatively, Au-NPs already functionalized with oligonucleotides, but lacking a fluorophore, were backfilled with Cy5-PEG-thiol (Nanocs). In this approach DNA Au-NPs were incubated with Cy5-PEG-thiol 30 μM for 4 hours at room temperature prior to purification by centrifugation at 14,000 rpm for 25 minutes x 3 and suspension in PBS.

### Dasatinib-oligonucleotide conjugation

The 5′-alkynyl modified deoxyoligonucleotides (**3**; Supplementary Figure 1) were ordered from IDT or prepared with an Expedite nucleic acid synthesizer using a 5′-hexynyl phosphoramidite. The dasatinib azido derivative **2** (Supplementary Figure 1) was prepared from commercially available dasatinib (**1**; Supplementary Figure 1) according to a literature procedure with slight modifications [30]. To the mixture of dasatinib (488 mg, 1.0 mmol) in dry DMF/THF (15 mL, 2:1, v/v) at 0°C, triethylamine (0.28 mL, 2.0 mmol) was added and the mixture was stirred for 10 minutes. Methanesulfonyl chloride (155 μL, 2.0 mmol) was added slowly and the solution was allowed to warm up to room temperature overnight. Sodium azide (152 mg, 2.33 mmol) was added and the mixture was heated to 50°C and stirred for 47 hrs. After cooling to room temperature, about 5 mL reaction mixture (~1/3 portion, 0.33 mmol) was taken out, quenched with water, and extracted with dichloromethane. The organic layers were combined. The solvent was removed, the residue was isolated by silica gel chromatography, eluting with 5% methanol in dichloromethane to yield **2** (Supplementary Figure 1) as a white solid 139 mg (82% yield). MS (ES-API) calculated for C_22_H_26_ClN_10_OS [MH^+^] 513.2, found 513.2.

To a solution of 5′-alkynyl oligonucleotide **3** (71 nmol) in water (200 μL), were added **2** (Supplementary Figure 1) in DMSO (355 μL, 10 mM, 3.55 μmol), THPTA (tris(3-hydroxypropyltriazolylmethyl)amine)/CuSO4 (1:1) complex in water (27 μL, 66 mM, 1.78 μmol), and aqueous sodium ascorbate (28.4 μL, 100 mM, 2.84 μmol). The mixture was degassed with argon. After the mixture was kept at room temperature for 1 hour and chloroform extracted, the aqueous phase was loaded onto a 20% dPAGE gel and purified. Dasatinib-DNA conjugate (**I**; Supplementary Figure 1) was obtained in 33% yield (23.6 nmol) and MALDI-Tof MS calculated for MH^+^, 5008.0, found 5007.1. Dasatinib-DNA conjugate (**II**; Supplementary Figure 1) was obtained in 32% yield (64 nmol) and MALDI-Tof MS calculated for MH^+^, 5287.3, found 5286.4.

### *In vitro* measurement of DNA-Au NP sequence specificity

Cy5-DNA conjugated gold NPs were incubated in PBS at a concentration of 1 nM. An excess of fully complementary, or scrambled control, oligonucleotide was added to the solution. The mixture was allowed to incubate at room temperature for 30 minutes, after which the resulting fluorescence was measured from 640-740 nm with a Molecular Devices SpectraMax plate reader with an excitation of 610 nm and cut-off of 665 nm.

### RT-PCR

Leukemia cells were treated with DNA-Au NPs (5 nM) for 96 hours. The locked nucleic acid (LNA) Au-NPs contained 4 LNAs at both the 5′ and 3′ termini (**CCCA**GCCTTCCAGCTCC**TTGT**_10_; bold denotes LNAs). Total RNA was isolated using the RNeasy Plus mini kit (Quiagen). Reverse transcription was performed with 1 μg total RNA and qScript cDNA Supermix (Quanta Biosciences). Quantitative real time PCR was performed using PerfeCTa® SYBR® Green SuperMix (Quanta Biosciences) on an ABI 7500 PCR system. The primers used were 5′- AGGACCACCGCATCTCTACAT-3′ (sense) and 5′-GTTCTGAGTGTGACCGAGAAGGTA-3′ (antisense) for *BIRC5* and 5′- AAGTCTGGCTCGTTCTCAGTG −3′ (sense) and 5′-CAGGAAAGACACCCACCTTGATCT-3′ (antisense) for β-*actin*. All reactions were run in triplicate, and the relative expression of *BIRC5* was calculated by normalizing *BIRC5* mRNA expression to β-*actin* mRNA expression.

### *In vitro* kinase assays

Src and c-KIT *in vitro* ADP-Glo Kinase Assays (Promega) were used according to manufacturer's instructions. Luminescence was measured in the presence of increasing concentrations of either dasatinib or dasatinib-conjugated oligonucleotide and 100 ng of recombinant enzyme (Promega). IC50 values were calculated using GraphPad Prism 6.0 and nonlinear regression log(inhibitor) vs. response model. Alternatively, luminescence was measured in the presence of dasatinib-DNA Au-NPs and an excess of either a scrambled oligonucleotide or an oligonucleotide mimicking the sequence of the *BIRC5* mRNA.

### Cell lines and culture

Leukemia cell lines were obtained from ATCC and DSMZ. Leukemia cell lines were grown in RPMI supplemented with FCS 10% and penicillin-streptomycin. The media for SKNO-1 also included GM-CSF 10 ng/mL. The media for 32D cells included murine IL-3 1 ng/mL. HEK293T and 3T3 cells were grown in DMEM supplemented with FCS 10% and penicillin-streptomycin. GFP expressing K562 leukemia cells were generated by transducing K562 cells with MSCV PIG (Puro-IRES-GFP; Addgene plasmid #18751) retrovirus and selecting with puromycin.

### Generation of BIRC5, and AML1-ETO mRNA inducible cell lines

AML1/ETO was PCR amplified using Q5 High-Fidelity Polymerase (New England Biolabs) from a pMSCV-AML1/ETO plasmid (gift from Dr. J. Mulloy). The PCR product was transferred into the EcoRI and AgeI sites of the tetracycline-inducible pLVX-Tet-One vector (Clontech) using In-Fusion cloning (Clontech). A portion of the resulting pLVX-Tet-One vector was then PCR amplified (Forward Primer: 5′- CAGCAGAGATCCAGTTTATCGACTT-3′; Reverse Primer: 5′- TGCAGAATTAATTCCAGGCGGG -3′) and transferred into a plasmid with AAVS1 homology arms (derived from AAVS1-SA-2A-puro-pA donor, Addgene plasmid #22075) using In-fusion cloning. The inducible, transgene plasmids were then co-transfected into HEK293T cells using Lipofectamine 3000 (Thermo Fisher) along with the gRNA_AAVS1-T2 (Addgene plasmid #41818) and pCas9_GFP (Addgene Plasmid #44719) plasmids. Three days after transfection the resistant cells were selected with puromycin (2 μg/mL). For the *BIRC5* gene, the human coding sequence was obtained as GeneArt Gene Synthesis (Invitrogen) and cloned into the EcoRI and BamHI site of the pLVX-Tet-One vector (Clontech) using the In-Fusion HD cloning system (Clontech). This vector was then used to generate lentiviral particles that were used to transduce NIH3T3 cells. After cells were selected with puromycin, doxycycline (1 μg/mL)-inducible *BIRC5* mRNA expression was confirmed by quantitative RT-PCR.

### Microscopy

Cells were grown on glass bottom, tissue culture plates (MatTek) in the presence or absence of doxycycline 1 μg/mL. After 48 hours of culture in doxycycline, Cy5-DNA Au-NPs (0.5 nM) were added to the cells. After overnight incubation, the cells were stained with Hoechst 33342 (1 μg/mL) for 15 minutes, washed, and assessed by microscopy with an EVOS FL Auto Imaging System (Life Technologies).

### Flow cytometry assays

Antibodies against phospho-SRC (clone SC1T2M3), phospho-Akt (clone SDRNR), CD69 (clone FN50), CD4 (clone SK3), CD8 (clone SK3), and isotype controls were from eBiosciences. Phospho-CrkL (clone K30-391.50.80) was from BD Biosciences. Cells were treated as described and then fixed, permeabilized, and stained with antibodies for 30-60 minutes in the dark. The cells were analyzed by flow cytometry after washing on a BD FACSAria II.

### Proliferation, apotosis, and colony assays

In 96-well plate format, leukemia cells were treated as described for 48 hours and then viability assessed with the CellTiter-Glo Luminescent Cell Viability Assay (Promega). All experiments were performed in triplicate with at least 3 wells per condition. To assess apoptosis, leukemia cells were treated with dasatinib or dasatinib-DNA Au-NPs for 72 hours, stained with annexin-V, and analyzed by flow cytometry. For colony-formation assays, 10^3^ K562 cells were plated in methylcellulose (Stem Cell Technologies) following 4 hours of pre-treatment with Au-NPs (1 nM). Colonies were counted 7 days after plating.

### Competition assays

Either murine bone marrow cells (red cell lysed) or human bone marrow mononuclear cells (Lonza) were mixed at specified ratios with leukemia cells stained with Cell-Trace Violet (Invitrogen). Cy5-DNA Au-NPs (Cy5 covalently attached to Au-NP as described above; 0.5 nM) were then added to the cells and allowed to incubate overnight. The cells were then analyzed by flow cytometry. Within the violet gate (leukemia cells), the percent of cells positive for Cy5 was measured. For *in vivo* experiments, GFP expressing K562 leukemia cells were injected subcutaneously into both flanks of NSG mice. When tumors became palpable (~10-14 days post-injection), Cy5-DNA Au-NPs were injected directly into a tumor on one flank. The next day, the mouse was euthanized, both tumors excised, dissociated by passing through a 40 micron cell strainer, and analyzed by flow cytometry as described above.

### CD34 and T-cell assays

Freshly defrosted human CD34 cells (Lonza) were grown in cytokine containing media (Stem Cell Factor 100 ng/mL, Thrombopoietin 100 ng/mL, and FLT3 ligand 100 ng/mL) for 24 hours prior to the addition of dasatinib 5 nM or dasatinib-DNA Au-NPs 5 nM. After an additional 24 hour incubation, the CD34 cells were fixed and permeabilized as described above, stained for phospho-Src, and analyzed by flow cytometry. Human CD3 T-cells (Astarte Biologics) were grown in 96 wells plates (80,000 cells/well). T-cells were then treated for 6 hours with 5 nM dasatinib, dasatinib-DNA Au-NPs, or control Au-NPs prior to being stimulated by the addition of 2 μL of CD3/CD28 Dynabeads, according to manufacturer's instructions (Invitrogen). After 24 hours, the T-cell/bead mix was vortexed and thoroughly pipetted prior to removing the beads by placing the mixture in a magnet for 2 minutes and then removing the supernatant containing cells. The T-cells were then stained for CD69 and analyzed by flow cytometry. To assess Au-NP uptake, 100,000 CD34 cells and T-cells were treated overnight with 0.5 nM Au-NPs covalently labeled with Cy5. The following day, the percent cells Cy5 positive was determined by flow cytometry. Prior to flow cytometry, the T-cells were also stained for CD4 and CD8.

### Xenograft studies

NSG (NOD.Cg-*Prkdcscid, Il2rgtm1Wjl*/SzJ; Jackson Labs) mice were housed under aseptic conditions and received autoclaved cages, bedding material, water, bottles, and irradiated food. Mouse care was in accordance with protocols approved by the Institutional Animal Care and Use Committee at the University of Minnesota. K562 leukemia cells were treated for 4 hours with dasatinib-DNA Au-NPs 1 nM or control DNA Au-NPs lacking dasatinib, prior to being injected intravenously via the tail vein into 5-7 week old NSG mice. Mice were observed until death or they met euthanasia criteria secondary to leukemia.

### Statistical analysis

Results are shown as the mean plus or minus the SEM of the results of at least 3 experiments. The Student's *t*-test or ANOVA were used for statistical comparisons between groups and were calculated using GraphPad Prism 6 software (GraphPad Software, La Jolla, CA). For the mouse survival curve a log-rank test was calculated. *P*-values less than 0.05 were considered statistically significant.

## SUPPLEMENTARY FIGURES AND TABLES



## References

[1] Barnaby SN, Sita TL, Petrosko SH, Stegh AH, Mirkin CA (2015). Therapeutic applications of spherical nucleic acids. Cancer Treat Res.

[2] Rosi NL, Giljohann DA, Thaxton CS, Lytton-Jean AKR, Han MS, Mirkin CA (2006). Oligonucleotide-modified gold nanoparticles for intracellular gene regulation. Science.

[3] Prigodich AE, Seferos DS, Massich MD, Giljohann DA, Lane BC, Mirkin CA (2009). Nano-flares for mRNA regulation and detection. ACS Nano.

[4] Prigodich AE, Randeria PS, Briley WE, Kim NJ, Daniel WL, Giljohann DA, Mirkin CA (2012). Multiplexed nanoflares: mRNA detection in live cells. Anal Chem.

[5] Giljohann DA, Seferos DS, Daniel WL, Massich MD, Patel PC, Mirkin CA (2010). Gold nanoparticles for biology and medicine. Angew Chem Int Ed Engl.

[6] Jensen SA, Day ES, Ko CH, Hurley LA, Luciano JP, Kouri FM, Merkel TJ, Luthi AJ, Patel PC, Cutler JI, Daniel WL, Scott AW, Rotz MW (2013). Spherical nucleic acid nanoparticle conjugates as an RNAi-based therapy for glioblastoma. Sci Transl Med.

[7] Bao C, Conde J, Curtin J, Artzi N, Tian F, Cui D (2015). Bioresponsive antisense DNA gold nanobeacons as a hybrid *in vivo* theranostics platform for the inhibition of cancer cells and metastasis. Sci Rep.

[8] Lindauer M, Hochhaus A (2014). Dasatinib. Recent Results Cancer Res.

[9] Rix U, Hantschel O, Dürnberger G, Remsing Rix LL, Planyavsky M, Fernbach NV, Kaupe I, Bennett KL, Valent P, Colinge J, Köcher T, Superti-Furga G (2007). Chemical proteomic profiles of the BCR-ABL inhibitors imatinib, nilotinib, and dasatinib reveal novel kinase and nonkinase targets. Blood.

[10] Avti PK, Maysinger D, Kakkar A (2013). Alkyne-azide “click” chemistry in designing nanocarriers for applications in biology. Molecules.

[11] Cheung CHA, Huang C-C, Tsai F-Y, Lee JY-C, Cheng SM, Chang Y-C, Huang YC, Chen SH, Chang JY (2013). Survivin - biology and potential as a therapeutic target in oncology. Onco Targets Ther.

[12] Fröhling S, Döhner H (2008). Chromosomal abnormalities in cancer. N Engl J Med.

[13] Oda T, Heaney C, Hagopian JR, Okuda K, Griffin JD, Druker BJ (1994). Crkl is the major tyrosine-phosphorylated protein in neutrophils from patients with chronic myelogenous leukemia. J Biol Chem.

[14] Larizza L, Magnani I, Beghini A (2005). The Kasumi-1 cell line: a t(8;21)-kit mutant model for acute myeloid leukemia. Leuk Lymphoma.

[15] Balkhi MY, Christopeit M, Chen Y, Geletu M, Behre G (2008). AML1/ETO-induced survivin expression inhibits transcriptional regulation of myeloid differentiation. Exp Hematol.

[16] Mpakou VE, Kontsioti F, Papageorgiou S, Spathis A, Kottaridi C, Girkas K, Karakitsos P, Dimitriadis G, Dervenoulas I, Pappa V (2013). Dasatinib inhibits proliferation and induces apoptosis in the KASUMI-1 cell line bearing the t(8;21)(q22;q22) and the N822K c-kit mutation. Leuk Res.

[17] Seferos DS, Giljohann DA, Rosi NL, Mirkin CA (2007). Locked nucleic acid-nanoparticle conjugates. Chembiochem.

[18] Han L, Schuringa JJ, Mulder A, Vellenga E (2010). Dasatinib impairs long-term expansion of leukemic progenitors in a subset of acute myeloid leukemia cases. Ann Hematol.

[19] Schade AE, Schieven GL, Townsend R, Jankowska AM, Susulic V, Zhang R, Szpurka H, Maciejewski JP (2008). Dasatinib, a small-molecule protein tyrosine kinase inhibitor, inhibits T-cell activation and proliferation. Blood.

[20] Blake S, Hughes TP, Mayrhofer G, Lyons AB (2008). The Src/ABL kinase inhibitor dasatinib (BMS-354825) inhibits function of normal human T-lymphocytes *in vitro*. Clin Immunol.

[21] Blake SJ, Bruce Lyons A, Fraser CK, Hayball JD, Hughes TP (2008). Dasatinib suppresses *in vitro* natural killer cell cytotoxicity. Blood.

[22] Gratacap M-P, Martin V, Valéra M-C, Allart S, Garcia C, Sié P, Recher C, Payrastre B (2009). The new tyrosine-kinase inhibitor and anticancer drug dasatinib reversibly affects platelet activation *in vitro* and *in vivo*. Blood.

[23] Futosi K, Németh T, Pick R, Vántus T, Walzog B, Mócsai A (2012). Dasatinib inhibits proinflammatory functions of mature human neutrophils. Blood.

[24] Vandyke K, Dewar AL, Diamond P, Fitter S, Schultz CG, Sims NA, Zannettino AC (2010). The tyrosine kinase inhibitor dasatinib dysregulates bone remodeling through inhibition of osteoclasts *in vivo*. J Bone Miner Res.

[25] Rix U, Superti-Furga G (2009). Target profiling of small molecules by chemical proteomics. Nat Chem Biol.

[26] Zhang K, Hao L, Hurst SJ, Mirkin CA (2012). Antibody-linked spherical nucleic acids for cellular targeting. J Am Chem Soc.

[27] Benedetto G, Vestal CG, Richardson C (2015). Aptamer-Functionalized Nanoparticles as “Smart Bombs”: The Unrealized Potential for Personalized Medicine and Targeted Cancer Treatment. Target Oncol.

[28] Lane LA, Qian X, Smith AM, Nie S (2015). Physical chemistry of nanomedicine: understanding the complex behaviors of nanoparticles *in vivo*. Annu Rev Phys Chem.

[29] Taton TA (2002). Preparation of gold nanoparticle-DNA conjugates. Curr Protoc Nucleic Acid Chem.

[30] Fischer JJ, Dalhoff C, Schrey AK, Graebner OY, Michaelis S, Andrich K, Glinski M, Kroll F, Sefkow M, Dreger M, Koester H (2011). Dasatinib, imatinib and staurosporine capture compounds - Complementary tools for the profiling of kinases by Capture Compound Mass Spectrometry (CCMS). J Proteomics.

