# Supplementary material for: Drug conjugated nanoparticles activated by cancer cell specific mRNA
Source: Oncotarget. 2016 May 18;7(25):38243–56. doi: 10.18632/oncotarget.9430 (PMC5122386; doi:10.18632/oncotarget.9430)
Supplement: Supplementary file 1 [file oncotarget-07-38243-s001.pdf]

Drug conjugated nanoparticles activated by cancer cell specific mRNA

SUPPLEMENTARY FIGURES AND TABLE

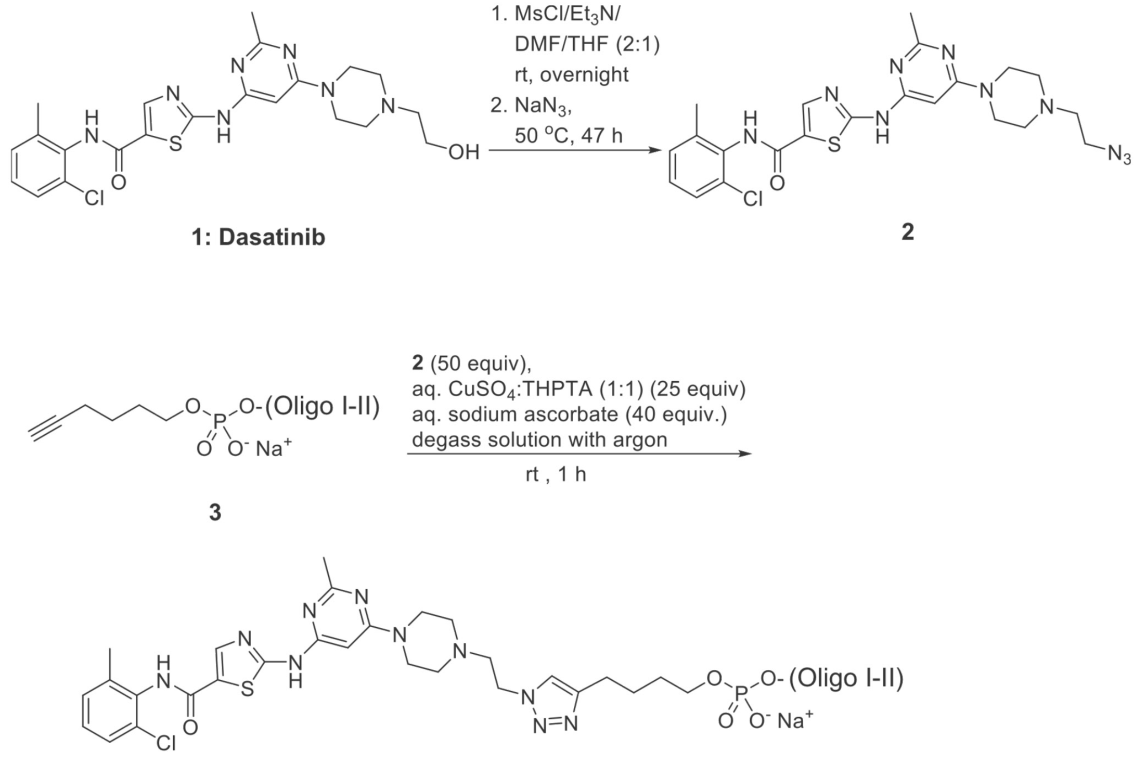

I (BIRC5): 5'-TCAAGGAGCTGG-3'
II (Scramble): 5'-CTTGGATGATCGGTT-3'

Supplementary Figure S1: Schematic showing the conjugation of dasatinib to an oligonucleotide. By utilizing "click chemistry" the azide modified dasatinib (**2**) can linked to any oligonucleotide containing a commercially available alkyne modification (**3**) to yield a dasatinib-conjugated oligonucleotide.

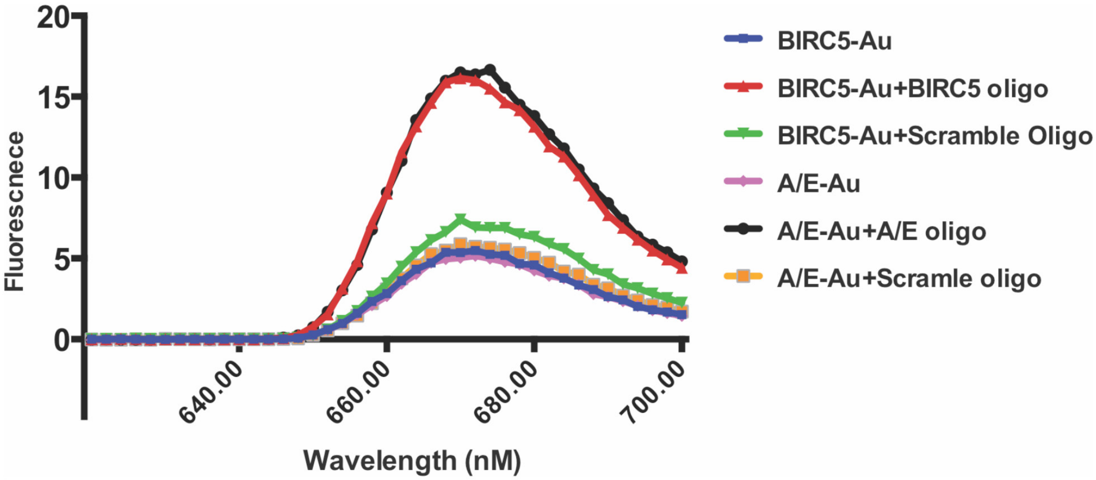

Supplementary Figure S2: *In vitro* specificity of Au-NPs. Cy5-DNA Au-NPs targeting the *BIRC5* mRNA or *AML1/ETO* breakpoint sequence were incubated with an excess of an oligonucleotide mimicking the targeted RNA sequence or a scrambled control oligonucleotide. After thirty minutes, fluorescence was measured using a fluorescence plate reader.

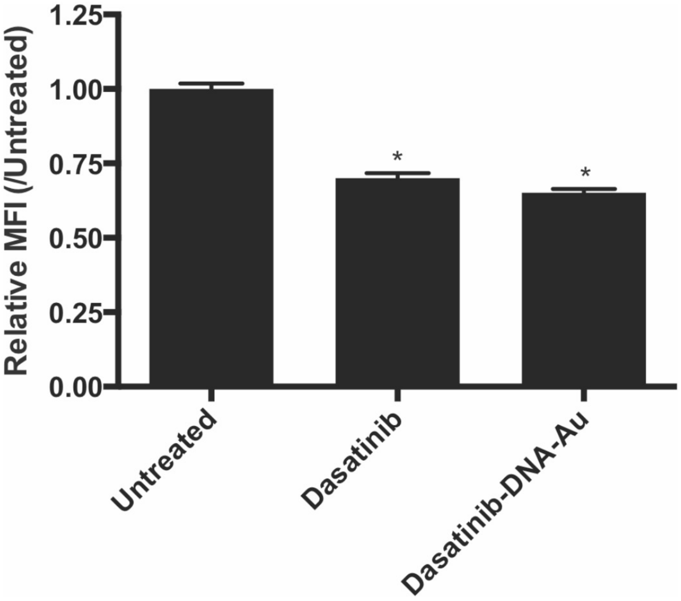

Supplementary Figure S3: Kasumi-1 leukemia cells were treated with dasatinib (10 nM) or dasatinib-DNA Au-NPs (1 nM) targeting the human *BIRC5* mRNA. After 24 hours the cells were fixed/permeabilized, stained for intracellular phospho-AKT, and assessed by flow cytometry. Median fluorescence intensity (MFI) for each condition relative to the untreated control was calculated. *, $P < 0.0001$ when comparing either dasatinib or dasatinib-DNA Au-NP treated cells with untreated control cells.

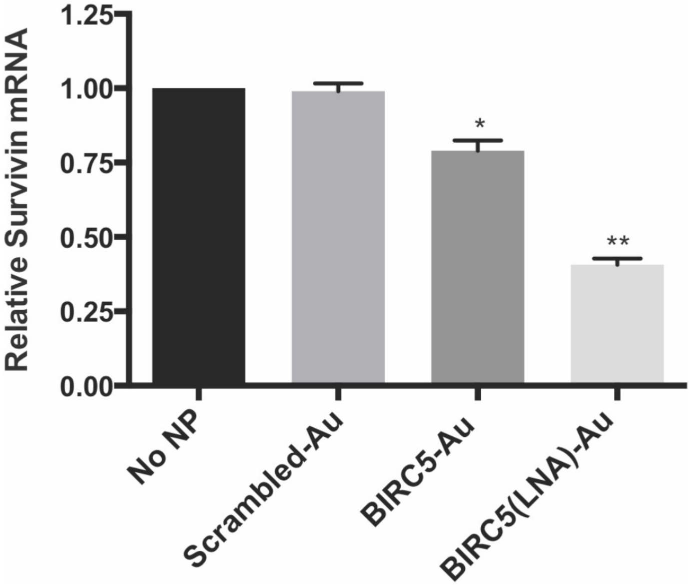

Supplementary Figure S4: *BIRC5* mRNA knockdown by DNA-conjugated Au-NPs. Quantitative reverse-transcriptase-PCR analysis of total RNA isolated from K562 cells after 96 hours of treatment with DNA Au-NPs targeting the human *BIRC5* mRNA. *BIRC5* mRNA levels were normalized to *β-actin* expression. *BIRC5* (LNA)-Au contains locked nucleic acids as described in methods. Reactions were performed in triplicate and the data are the mean±s.e.m. from three independent experiments. *, $P < 0.0005$ and **, $P < 0.0001$ when compared to untreated cells.

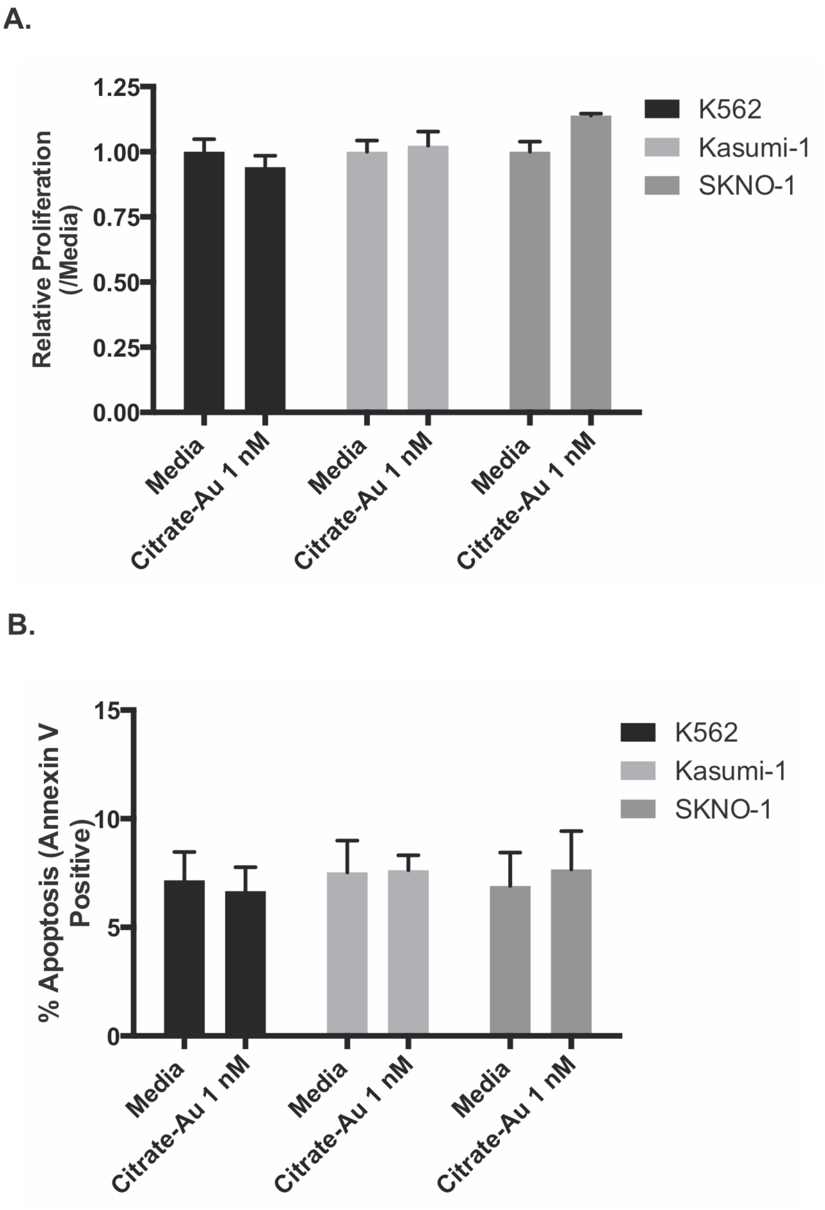

Supplementary Figure S5: Effect of citrate-capped Au-NPs on leukemia cell proliferation and apoptosis. K562, Kasumi-1, and SKNO-1 leukemia cells were treated with citrate-capped Au-NPs 1 nM for 72 hours and then proliferation **A.** and apoptosis **B.** were assessed using the CellTiter-Glo Viability Assay (Promega) and Annexin-V staining, respectively.

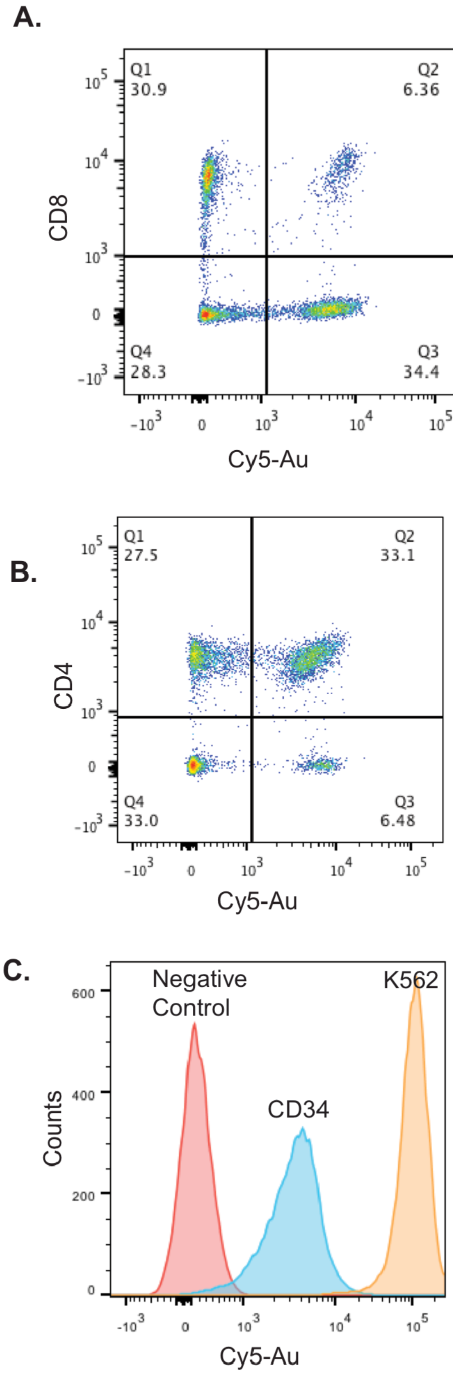

Supplementary Figure S6: Uptake of Au-NPs by CD34 cells and T-cells. Flow cytometry plots of human CD3 T-cells treated overnight with Au-NPs covalently labeled with Cy5 and then stained for CD8 **A.** and CD4 **B.** Percent cells are shown in each quadrant. **C.** Flow cytometry plot of human CD34 cells and K562 leukemia cells treated overnight with an equal amount of Au-NPs covalently labeled with Cy5.

Supplementary Table S1: Oligonucleotide sequences and modifications

Name	Sequence	5' Modification	3' Modification
BIRC5-thiol	5'-CCC AGC CTT CCA GCT CCT TGA AAA AAA AAA A-3'		Propylthiol
AML1/ETO-thiol	5'-CTT CTT CCA TTG CGA CCA AAA AAA-3'		Propylthiol
Scramble-thiol	5'-ACC ATT AAC CGA TCA TCC AAG AAA AAA AA-3'		Propylthiol
Scramble-Alkyne	5'-CTT GGA TGA TCG GTT-3'	Hexynyl	
BIRC5-alkyne	5'-TCA AGG AGC TGG-3'	Hexynyl	
BIRC5-Cy5	5'-TCA AGG AGC TGG-3'	Cy5	
AML1-ETO-Cy5	5'-CTC GAA ATC GTA-3'	Cy5	
Cy5-Uptake Control-thiol	5'-ATC GAA TTC CTG CAG CCC GTT-3'	Cy5	C18 Spacer-Propylthiol
BIRC5-mimic	5'-CAA GGA GCT GGA AGG CTG GG-3'		
AML1-ETO-mimic	5'-TCG AAA TCG TAC TGA GAA-3'		
